# Analysis of risk factors associated with pre-myopia among primary school students in the Mianyang Science City

**DOI:** 10.16910/jemr.17.1.3

**Published:** 2024-03-18

**Authors:** Yi-bin Deng, Xiao-yin Wang, Li-ge Xiao, Pei li Xu, Hui-Min Wang, Guo-Zhong Zhao, Lian Ye, Da-Wei Men, Mei Yan

**Affiliations:** Hospital of Chengdu University of Traditional Chinese Medicine Chengdu,Sichuan, China; Department of Clinical Research Management, West China Hospital, Sichuan University, Chengdu,Sichuan, China; Sichuan Science City Hospital Mianyang, Sichuan, China; Si Chuan Provincial Hospital for Women and Children Chengdu,Sichuan, China

**Keywords:** Pre-myopia, Risk factors, Primary school students

## Abstract

Objectives To find out the prevalence rate of pre-myopia among primary school students in the Mianyang Science City Area, analyze its related risk factors, and thus provide a reference for local authorities to formulate policies on the prevention and control of myopia for primary school students.

Methods From September to October 2021, Cluster sampling was adopted by our research group to obtain the vision levels of primary school students employing a diopter test in the Science City Area. In addition, questionnaires were distributed to help us find the risk factors associated with pre-myopia. Through the statistical analysis, we identify the main risk factors for pre-myopia and propose appropriate interventions.

Results The prevalence rate of pre-myopia among primary school students in the Science City Area was 45.27% (1020/2253), of which 43.82% were boys and 46.92% were girls, with no statistically significant difference in the prevalence rate of myopia between boys and girls (2 =2.171, P=0.141). The results of the linear trend test showed that the prevalence rate of pre-myopia tends to decrease with increasing age (Z=296.521, P=0.000). Logistic regression analysis demonstrated that the main risk factors for pre-myopia were having at least one parent with myopia, spending less than 2 hours a day outdoors, using the eyes continuously for more than 1 hour, looking at electronic screens for more than 2 hours, and having an improper reading and writing posture.

Conclusion The Science City Area has a high prevalence rate of pre-myopia among primary school students. It is proposed that students, schools, families, and local authorities work together to increase the time spent outdoors, reduce digital screens and develop scientific use of eye habits.

## Introduction

Pre-myopia is a period that precedes myopia and is a non-myopic refractive state (1). However, it has a high potential to develop into shortsightedness. Analyzing the relevant risk factors for pre-myopia, and adopting appropriate interventions to address these factors could prevent or delay the onset of myopia, which is particularly important for myopia prevention and control. Furthermore, it is also an effective means to implement the Comprehensive Plan to Prevent Shortsightedness among Children (National Radio and Television Administration. 2018) jointly issued by eight national ministries and departments, which is the key to successful myopia prevention and control. From September to October 2021, our research group conducted an eye test to survey the risk factors associated with pre-myopia among primary school students in the Science City Area, and the results are as follows.

## Methods

### General information

Cluster sampling was used to conduct an eye test and questionnaires were dis-tributed to primary school students by Our research group in the Science City area from September to October 2021. The study was approved by the Ethics Committee of Sichuan Science City Hospital, and by excluding incomplete data, we finally obtained data from 2253 students (1198 boys and 1055 girls). The number of children in each age group, as well as the distribution of boys and girls in each age group were in Table 1.

### Quality Control

People participating in this eye test were ophthalmologists from Sichuan Science City Hospital. Before conducting the test in the school, we provided centralized and unified training so the staff was proficient in operating various instruments and testing principles, standardizing the testing procedures, unifying the testing methods, steps, criteria for determining each data, and recording methods and formats. The diopter test was performed using the TOPCON KR-800 Auto Ref/Keratometer. The instrument was calibrated and maintained before the formal test to maintain the accuracy of the measuring instrument. Questionnaires were completed by both par-ents and students during parents' meeting.

### Method for measuring diopter (Chinese Doctor Association. 2019).

Method for measuring diopter: Firstly, we made the student's lower jaw placed on the jaw rest and the forehead placed against the forehead rest. Following that, we adjusted the knob so that the corner of the student's eyes was at the same level as the height marker of the jaw test and instructed the student to look at the visual target. Next, our staff switched the keratometer into automatic measurement mode, holding the joystick and moving it towards the student to align the eyes in the center of the control panel. And then, people continued to slowly push the keratometer toward the student to make it match the focus of the student's eyes. Finally, the instrument automatically measured the diopter of the student and exported data to the spread-sheet.

### Evaluation Criteria

Pre-myopia is a condition in which the spherical equivalent of an eye is ≤ +0.75D and ＞-0.50D when ocular accommodation is relaxed (1).

Criteria for reading and writing posture (4): according to the "Programme for the Prevention and Control of Myopia among Primary and Secondary School Students" issued by the Ministry of Education in 2008, the evaluation criteria are the following three points: (1) the distance between the eyes and the book when reading and writing is 33-35cm (a foot), the distance between the table and the chest is about one's fist, and the distance be-tween the fingers holding the pen and the pen tip should be about 3cm (one inch); (2) the angle between the pencil and the paper surface when writing is between 40~50 degrees; (3) not walking to read, not lying down or tilting the head to read, not reading on a swaying car or boat. Any reading and writing postures above are considered incorrect.

### Data Entry and Data Processing

On the day of the physical examination, two staff were responsible for entering the data into Excel and performing statistical analysis through SPSS 25.0 after completing data collection. Regarding the data processing, the constituent ratios were expressed as percentages, the x2 test was adopted on comparison among groups, linear trend tests were performed using the Linear-by-Linear Association, and risk factors associated with pre-myopia were analyzed using Logistic regression analysis.

## Results

### Comparison of the prevalence rate of pre-myopia by sex and age

**Table 1.** Comparison of the prevalence rate of pre-myopia by sex and age [People（%]

Age (People)	Male/Female (People)	Pre-myopia		
		Male	Female	Overall
6(291)	133/158	66(49.62)	77(48.73)	143(49.14)
7(321)	150/171	83(55.33)	95(55.56)	178(55.45)
8(337)	183/154	116(63.39)	90(58.44)	206(61.13)
9(335)	163/172	96(58.90)	106(61.63)	202(60.30)
10(320)	178/142	63(35.39)	47(33.10)	110(34.38)
11(353)	193/160	60(31.09)	52(32.50)	112(31.73)
12(313)	161/152	41(25.47)	28(18.42)	69(22.04)
(2253)	1198/1055	525(43.82)	495(46.92)	1020(45.27)

The prevalence rate of pre-myopia was 45.27% (1020/2253), with 43.82% for males and 46.92% for females, and there was no statistically significant difference in the prevalence rate of pre-myopia between both sexes (x2 =2.171, P=0.141); the linear trend test showed a decreasing trend in the prevalence rate of pre-myopia with increasing age (Z=296.521, P=0.000). See Table 1.

### Analysis of risk factors associated with pre-myopia

Univariate analysis of risk factors associated with pre-myopia

With pre-myopia as the dependent variable (1=yes, 0=no) and other indicators as independent variables, the results demonstrated that at least one parent with severe myopia, at least one parent with myopia, the average time spent outdoors per day is below 2 hours in the last two weeks, continuous use of eyes over 1 hour, lack of sleep, the average time spent on digital screen per day over 2 hours, incorrect reading and writing postures, and the average time spent doing homework per day over 2 hours were the risk factors of pre-myopia. See Table 2.

**Table 2.** Univariate analysis of risk factors associated with pre-myopia [People(%)]

Variable	Grouping	Number of People	Constituent Ratio	Pre-myopia	Prevalence Rate	*x* ^2^	P
At Least One Parent with Severe Myopia	Yes	116	5.15	73	62.93	5.204	0.023
	No	2137	94.85	947	44.31		
At least one parent with myopia	Yes	1812	80.43	873	48.18	12.695	0.000
	No	441	19.57	147	33.33		
Average time spent outdoors per day below 2 hours in the last two weeks	Yes	302	13.40	178	58.94	9.187	0.002
	No	1951	86.60	842	43.16		
Continuous use of eyes over 1 hour	Yes	861	38.22	542	62.95	63.832	0.000
	No	1392	61.78	478	34.34		
Lack of sleep	Yes	217	9.63	132	60.83	8.073	0.004
	No	2036	90.37	888	43.61		
Average time spent on digital screen per day over 2 hours	Yes	309	13.72	205	66.34	21.609	0.000
	No	1944	86.28	815	41.92		
Incorrect reading and writing posture	Yes	1695	75.23	682	40.24	24.743	0.000
	No	558	24.77	338	60.57		
Average time spent doing homework per day over 2 hours	Yes	642	28.50	325	50.62	11.253	0.001
	No	1611	71.50	695	43.14		

### Multiple logistic regression analysis of risk factors associated with pre-myopia

The risk factors that were statistically significant in the univariate analysis were then conducted by multivariate analysis, with the presence of pre-myopia as the dependent variable (1=yes, 0=no) and other indicators as independent variables in a logistic regression analysis, which showed that at least one parent with myopia, time spent outdoors per day below 2 hours, continuous use of eyes over 1 hour, time spent on digital screen per day over 2 hours, and incorrect reading and writing postures were high-risk factors for developing pre-myopia. See Table 3.

**Table 3.** Logistic regression analysis of risk factors associated with pre-myopia

Influencing factor	β	SE	Waldχ2	P	OR	95%CI
At least one parent with myopia	0.64	0.043	65.31	<0.01	1.746	1.572-1.984
Time spent outdoors per day below 2 hours in the last two weeks	0.59	0.039	136.28	<0.01	1.827	1.668-2.032
Continuous use of eyes over 1 hour	0.43	0.056	40.37	<0.01	1.478	1.384-1.874
Time spent on digital screen per day over 2 hours	0.28	0.038	22.31	<0.01	1.583	1.468-1.892
Incorrect reading and writing posture	0.47	0.043	38.42	<0.01	1.672	1.487-1.924

## Discussion

A previous study has discussed that myopia is a combination of genetic and social factors, with behaviors and habits among the social factors playing a crucial role in the onset and development of myopia. (5) The primary school level is a significant period for developing good behaviors and habits, which is also the high incidence of pre-myopia. Another study has concluded that low concentrations of atropine can stop the progression of myopia by increasing the axial length (6), but it is prone to rebound after drug withdrawal. Hence, preventing the onset of myopia and reducing the prevalence rate of myopia is the key to the success of myopia prevention and control. Pre-myopia is a non-myopic refractive state and is a critical period for myopia prevention and control, which is likely to develop into myopia if left untreated. Our survey results showed that the prevalence rate of pre-myopia among primary school students in the Mianyang Science City Area is 45.27%, with a decreasing trend in the prevalence rate with age. There are studies that demonstrate the prevalence of myopia worldwide, depending on the region. Characteristically high prevalence rates of myopia in children have been found in East Asia, particularly in urban settings in populations of both East Asian and South Asian ethnicity(7).Associations of myopia in children include parental myopia, increased near work, and higher schooling. Parental myopia has been associated with a higher prevalence and a greater likelihood of childhood myopia in both European Caucasian and East Asian children(8).Given a considerable number of pre-myopia students, it is clear that analyzing the risk factors associated with pre-myopia during this period and targeting the right interventions to stop its progression could significantly reduce the prevalence rate, thus fulfilling the myopia prevention and control mandate from the eight national ministries and departments.

The results of the multivariate study showed that risk factors for pre-myopia are closely related to the following aspects:

(1) Genetic factors. Studies have shown that myopia is hereditary, either monogenic or polygenic. Children of nearsighted parents are significantly more likely to develop myopia (9), and children of nearsighted siblings are also more likely to develop myopia(10). The results of our study show that children of parents with myopia are at a significantly increased risk of developing pre-myopia, suggesting that pre-myopia might be genetically related and is at high risk of developing myopia if left untreated during this period.

(2) Outdoor time: a Meta-analysis on myopia (11) showed that outdoor activity can effectively improve students' dynamic visual acuity and uncorrected visual acuity(UCCA), and the time they spend outside was obviously negatively correlated with the prevalence of myopia: the longer the outdoor activity time the lower the prevalence of myopia (12)., and extending outdoor activity time also slowed down the development of myopia (13). Multivariate statistical analysis showed that a daily outdoor activity time shorter than 2 hours was a risk factor for the development of pre-myopia, which might be associated with the fact that UV light during outdoor activity inhibits the extension of axial length while increasing the release of retinal dopamine and decreasing the axial elongation of the eye(14). Therefore, primary school students are recommended to increase their daily outdoor time.

(3) Continuous use of eyes: Near vision work leads to retinal defocus, and to make the retinal imaging clear, the eye muscles automatically move the focus back behind the retina, making the choroid thinner. Excessive and continuous use of the eyes will cause the extension of axial length and following early pre-myopia. If such behavior is not intervened, it can lead to the focus of the posterior segment of the eye to move back, then present myopia (15). The results of this study show that continuous use of eyes for more than 1 hour is a high-risk factor for pre-myopia.

(4) Digital screens: In recent years, with the prevalence of electronic products, especially smartphones and online courses, students spend more time on games and social media, such as TikTok, WeChat, and QQ. Besides, schools assign homework through the Internetresults in students spending significantly increasing digital screens like smartphones and computers than before. One study found that the average time spent on digital screens by primary school students in Shanghai was 2 hours per day at school and significantly rose to 5 hours during holidays (16). .A survey from Taiwan revealed that students spent an average of 3.5 hours per day looking at digital screens(17)., significantly exceeding the recommendation issued by the American Academy of Pediatrics, in which students spend below 2 hours per day on digital screens. Our findings showed that in the Science City Area, the percentage of primary school students who spent on digital screens over 2 hours per day on average was 13.72%. When looking at digital screens, the pupils are constantly changing, which keeps the ciliary muscles tense and not well relaxed, resulting in hyperflexion of the lens and developing into pre-myopia. Furthermore, the results found that excessive screen time is a risk factor for pre-myopia, and it is recommended that home-school cooperation to reduce the amount of time students spend staring at screens.

(5) Incorrect reading and writing postures: Inappropriate reading and writing posture can affect the body shape and lead to scoliosis and are closely related to vision(18). Studies show that the proportion of primary school students with incorrect reading and writing postures is more than 70% (19; 20). Our survey finds that in the Science City Area, the proportion of primary school students who read and write improperly is as high as 75.23%, and thus urgent attention ought to be paid by parents and schools. Such incorrect postures are commonly associated with a short reading and writing distance, resulting in the extension of axial length (21). and pre-myopia, so correcting students' reading and writing postures is one of the most important ways to prevent myopia, which requires the full cooperation and efforts of schools and parents.

In summary, the prevalence rate of pre-myopia among primary school students in the area is high. Parents with myopia, short outdoor time, incorrect reading and writing postures, continuous use of eyes, and long time digital screens are all high-risk factors for pre-myopia. To effectively implement myopia prevention and control into practice and reduce the prevalence of myopia, it is recommended that students, schools, families, and relevant departments work together to enable youngsters to develop scientific eye hygiene habits.

### Ethics and Conflict of Interest

The authors declare that the contents of the article are in agreement with the ethics described in http://biblio.unibe.ch/portale/elibrary/BOP/jemr/ethics.html and that there is no conflict of interest regarding the publication of this paper.

### Acknowledgements

This research was supported in part by Mianyang Health Commission Foundation.
